# Supplementary material for: Effect of seasonality on chemical profile and antifungal activity of essential oil isolated from leaves Psidium salutare (Kunth) O. Berg
Source: PeerJ. 2018 Nov 1;6:e5476. doi: 10.7717/peerj.5476 (PMC6215697; doi:10.7717/peerj.5476)
Supplement: Data S1 [file peerj-06-5476-s001.docx]

Figure 1 A

|  | Concentraçao | Fluconazole | | | | AP1 | | | | AP2 | | | | AP3 | | | |
| --- | --- | --- | --- | --- | --- | --- | --- | --- | --- | --- | --- | --- | --- | --- | --- | --- | --- |
| 0 | 0.000000 | 100.000000 | 100.000000 | 100.000000 | 100.000000 | 100.000000 | 100.000000 | 100.000000 | 100.000000 | 100.000000 | 100.000000 | 100.000000 | 100.000000 | 100.000000 | 100.000000 | 100.000000 | 100.000000 |
| 8 | 0.903090 | 70.744000 | 70.744000 | 70.744000 | 70.744000 | 75.970000 | 74.460000 | 63.450000 | 71.290000 | 95.610000 | 79.850000 | 81.730000 | 100.060000 | 75.860000 | 96.930000 | 87.660000 | 77.550000 |
| 16 | 1.204120 | 50.978000 | 50.978000 | 50.978000 | 50.978000 | 76.250000 | 87.120000 | 75.010000 | 84.920000 | 100.300000 | 67.580000 | 77.520000 | 100.300000 | 91.950000 | 87.240000 | 88.580000 | 74.600000 |
| 32 | 1.505150 | 30.178000 | 30.178000 | 30.178000 | 30.178000 | 75.210000 | 73.560000 | 73.430000 | 83.610000 | 92.210000 | 80.040000 | 55.030000 | 99.060000 | 82.940000 | 88.500000 | 91.360000 | 81.420000 |
| 64 | 1.806180 | 30.567000 | 30.567000 | 30.567000 | 30.567000 | 67.730000 | 68.280000 | 61.130000 | 72.000000 | 88.220000 | 84.280000 | 90.450000 | 75.890000 | 84.140000 | 86.670000 | 80.770000 | 79.930000 |
| 128 | 2.107210 | 18.389000 | 18.389000 | 18.389000 | 18.389000 | 78.930000 | 74.800000 | 69.440000 | 76.180000 | 89.370000 | 84.350000 | 95.480000 | 88.290000 | 72.220000 | 72.390000 | 96.990000 | 71.710000 |
| 256 | 2.408240 | 23.011000 | 23.011000 | 23.011000 | 23.011000 | 67.180000 | 70.070000 | 60.030000 | 74.340000 | 74.970000 | 76.920000 | 81.710000 | 78.800000 | 78.690000 | 72.450000 | 90.310000 | 71.780000 |
| 512 | 2.709270 | 14.100000 | 14.100000 | 14.100000 | 14.100000 | 54.850000 | 73.980000 | 54.580000 | 73.980000 | 70.490000 | 71.350000 | 80.770000 | 44.280000 | 71.520000 | 66.300000 | 83.820000 | 79.440000 |
| 1.024 | 3.010300 | 4.022000 | 4.022000 | 4.022000 | 4.022000 | 28.640000 | 48.590000 | 38.270000 | 44.460000 | 69.910000 | 65.630000 | 75.050000 | 68.890000 | 54.470000 | 52.440000 | 66.600000 | 55.140000 |
| 2048 | 3.311330 | 0.000000 | 0.000000 | 0.000000 | 0.000000 | 10.410000 | 34.210000 | 7.790000 | 17.560000 | 7.260000 | 18.050000 | 28.500000 | 30.900000 | 2.490000 | 12.260000 | 22.200000 | 17.650000 |
| 4096 | 3.612360 | 0.000000 | 0.000000 | 0.000000 | 0.000000 | 11.520000 | 9.050000 | 8.080000 | 3.680000 | 0.000000 | 2.780000 | 0.000000 | 6.380000 | 15.160000 | 2.860000 | 8.930000 | 10.110000 |
| 8182 | 3.912859 | 0.000000 | 0.000000 | 0.000000 | 0.000000 | 0.000000 | 6.400000 | 5.570000 | 4.060000 | 0.000000 | 11.670000 | 6.360000 | 7.560000 | 28.010000 | 20.600000 | 20.770000 | 11.840000 |

Figure 1B

|  | Concentraçao | Fluconazole (IC_50_ = 16.8 μg/mL) | | | | AP1 (IC_50_ = 2.7 μg/mL) | | | | AP2 (IC_50_ = 8.0 μg/mL) | | | | AP3 (IC_50_ = 6.3 μg/mL) | | | |
| --- | --- | --- | --- | --- | --- | --- | --- | --- | --- | --- | --- | --- | --- | --- | --- | --- | --- |
| 0 | 0.000000 | 100.000000 | 100.000000 | 100.000000 | 100.000000 | 100.00 | 85.31 | 97.28 | 100.00 | 105.02 | 98.46 | 92.50 | 92.50 | 100.65 | 105.22 | 97.66 | 92.69 |
| 8 | 0.903090 | 70.744000 | 70.744000 | 70.744000 | 70.744000 | 31.19 | 18.30 | 17.99 | 22.49 | 55.91 | 84.15 | 11.98 | 81.56 | 82.46 | 20.23 | 23.21 | 34.54 |
| 16 | 1.204120 | 50.978000 | 50.978000 | 50.978000 | 50.978000 | 17.53 | 7.40 | 16.92 | 13.95 | 43.44 | 32.90 | 11.43 | 19.78 | 14.36 | 32.26 | 16.55 | 19.53 |
| 32 | 1.505150 | 30.178000 | 30.178000 | 30.178000 | 30.178000 | 5.26 | 2.95 | 0.00 | 5.56 | 19.04 | 13.47 | 3.93 | 6.91 | 10.98 | 12.38 | 5.82 | 9.39 |
| 64 | 1.806180 | 30.567000 | 30.567000 | 30.567000 | 30.567000 | 1.27 | 0.19 | 0.00 | 0.50 | 15.56 | 6.81 | 2.63 | 0.25 | 1.39 | 14.51 | 0.00 | 2.98 |
| 128 | 2.107210 | 18.389000 | 18.389000 | 18.389000 | 18.389000 | 2.34 | 0.00 | 0.00 | 0.00 | 16.45 | 8.10 | 0.00 | 4.92 | 1.04 | 5.62 | 2.04 | 9.19 |
| 256 | 2.408240 | 23.011000 | 23.011000 | 23.011000 | 23.011000 | 0.69 | 0.00 | 0.00 | 0.00 | 4.67 | 2.09 | 2.09 | 0.00 | 0.00 | 7.50 | 8.10 | 9.69 |
| 512 | 2.709270 | 14.100000 | 14.100000 | 14.100000 | 14.100000 | 0.00 | 0.00 | 0.00 | 0.00 | 9.64 | 0.00 | 0.00 | 0.00 | 0.00 | 0.50 | 2.29 | 0.00 |
| 1.024 | 3.010300 | 4.022000 | 4.022000 | 4.022000 | 4.022000 | 0.00 | 0.00 | 0.00 | 0.00 | 9.74 | 0.00 | 0.00 | 0.00 | 4.37 | 0.00 | 4.57 | 5.96 |
| 2048 | 3.311330 | 0.000000 | 0.000000 | 0.000000 | 0.000000 | 0.00 | 0.00 | 0.00 | 0.00 | 5.42 | 0.00 | 0.00 | 0.00 | 0.00 | 0.00 | 0.00 | 0.00 |
| 4096 | 3.612360 | 0.000000 | 0.000000 | 0.000000 | 0.000000 | 0.00 | 0.00 | 0.00 | 0.00 | 0.00 | 0.00 | 0.00 | 0.00 | 0.00 | 0.00 | 0.00 | 0.00 |
| 8182 | 3.912859 | 0.000000 | 0.000000 | 0.000000 | 0.000000 | 0.00 | 0.00 | 0.00 | 0.00 | 0.00 | 0.00 | 0.00 | 0.00 | 0.00 | 0.00 | 0.00 | 0.00 |

Figure 1C

|  | Concentraçao | Fluconazole (IC_50_ = 9.0 μg/mL) | | | | AP1 (IC_50_ = 2570.4 μg/mL) | | | | AP2 (IC_50_ = 2754.2 μg/mL) | | | | AP3 (IC_50_ = 1621.8 μg/mL) | | | |
| --- | --- | --- | --- | --- | --- | --- | --- | --- | --- | --- | --- | --- | --- | --- | --- | --- | --- |
| 0 | 0.000000 | 100.000000 | 100.000000 | 100.000000 | 100.000000 | 100.00 | 100.00 | 100.00 | 100.00 | 100.00 | 100.00 | 100.00 | 100.00 | 100.00 | 100.00 | 100.00 | 100.00 |
| 8 | 0.903090 | 52.280000 | 52.280000 | 52.280000 | 52.280000 | 88.11 | 91.03 | 67.66 | 82.72 | 90.39 | 77.08 | 69.79 | 70.35 | 85.15 | 87.31 | 83.54 | 65.22 |
| 16 | 1.204120 | 49.468000 | 49.468000 | 49.468000 | 49.468000 | 94.72 | 88.11 | 66.58 | 85.80 | 91.28 | 87.64 | 83.99 | 82.31 | 79.02 | 71.75 | 84.28 | 80.91 |
| 32 | 1.505150 | 35.334000 | 35.334000 | 35.334000 | 35.334000 | 92.79 | 89.10 | 84.49 | 81.57 | 75.69 | 82.14 | 81.86 | 90.68 | 90.40 | 86.77 | 84.21 | 89.19 |
| 64 | 1.806180 | 24.544000 | 24.544000 | 24.544000 | 24.544000 | 75.52 | 83.82 | 66.14 | 84.90 | 83.92 | 78.74 | 80.70 | 71.03 | 86.38 | 73.99 | 76.68 | 86.78 |
| 128 | 2.107210 | 31.497000 | 31.497000 | 31.497000 | 31.497000 | 91.56 | 81.26 | 88.64 | 64.05 | 72.77 | 92.10 | 84.96 | 74.31 | 95.44 | 66.21 | 75.77 | 88.43 |
| 256 | 2.408240 | 26.216000 | 26.216000 | 26.216000 | 26.216000 | 93.35 | 85.66 | 84.74 | 74.29 | 82.52 | 75.94 | 83.92 | 88.83 | 90.51 | 60.47 | 86.20 | 75.96 |
| 512 | 2.709270 | 34.119000 | 34.119000 | 34.119000 | 34.119000 | 92.03 | 80.96 | 76.81 | 71.27 | 84.13 | 83.01 | 79.65 | 65.36 | 82.49 | 63.23 | 68.75 | 81.14 |
| 1.024 | 3.010300 | 25.114000 | 25.114000 | 25.114000 | 25.114000 | 75.96 | 65.66 | 68.89 | 77.04 | 73.71 | 69.65 | 77.78 | 90.39 | -2.93 | 52.42 | 61.45 | 65.35 |
| 2048 | 3.311330 | 24.772000 | 24.772000 | 24.772000 | 24.772000 | 66.20 | 62.51 | 54.52 | 64.20 | 70.39 | 60.02 | 69.26 | 50.77 | 69.19 | 51.68 | 56.80 | 20.98 |
| 4096 | 3.612360 | 16.831000 | 16.831000 | 16.831000 | 16.831000 | 48.23 | 30.86 | 43.47 | 29.94 | 44.03 | 37.16 | 40.39 | 28.62 | 38.25 | 32.19 | 30.30 | 8.22 |
| 8182 | 3.912859 | 17.287000 | 17.287000 | 17.287000 | 17.287000 | 2.69 | 0.23 | 0.00 | 0.00 | 10.39 | 5.20 | 1.98 | 2.82 | 8.38 | 4.88 | 6.36 | 6.63 |

Figure 1D

|  | Concentraçao | Fluconazole (IC_50_ = 9.3 μg/mL) | | | | AP1 (IC_50_ = 2.6 μg/mL) | | | | AP2 (IC_50_ = 5.3 μg/mL) | | | | AP3 (IC_50_ = 3.7 μg/mL) | | | |
| --- | --- | --- | --- | --- | --- | --- | --- | --- | --- | --- | --- | --- | --- | --- | --- | --- | --- |
| 0 | 0.000000 | 100.000000 | 100.000000 | 100.000000 | 100.000000 | 100.00 | 85.31 | 97.28 | 100.00 | 100.00 | 100.00 | 96.46 | 96.46 | 100.00 | 100.00 | 98.89 | 94.92 |
| 8 | 0.903090 | 52.280000 | 52.280000 | 52.280000 | 52.280000 | 25.93 | 13.04 | 12.73 | 0.46 | 42.86 | 64.50 | 56.62 | 62.51 | 65.83 | 16.15 | 18.53 | 27.58 |
| 16 | 1.204120 | 49.468000 | 49.468000 | 49.468000 | 49.468000 | 12.27 | 2.15 | 11.66 | 0.00 | 33.30 | 25.22 | 8.76 | 15.16 | 11.47 | 25.75 | 13.21 | 15.60 |
| 32 | 1.505150 | 35.334000 | 35.334000 | 35.334000 | 35.334000 | 3.38 | 1.07 | 0.00 | 0.00 | 14.59 | 10.32 | 3.01 | 5.30 | 8.77 | 9.88 | 4.64 | 7.50 |
| 64 | 1.806180 | 24.544000 | 24.544000 | 24.544000 | 24.544000 | 1.27 | 0.00 | 0.00 | 0.00 | 11.92 | 5.22 | 2.02 | 6.39 | 1.11 | 11.59 | 0.00 | 2.38 |
| 128 | 2.107210 | 31.497000 | 31.497000 | 31.497000 | 31.497000 | 2.34 | 0.00 | 0.00 | 0.00 | 12.61 | 6.21 | 0.00 | 3.77 | 0.83 | 4.48 | 0.00 | 7.34 |
| 256 | 2.408240 | 26.216000 | 26.216000 | 26.216000 | 26.216000 | 0.69 | 0.00 | 0.00 | 0.00 | 3.58 | 1.60 | 1.60 | 0.00 | 0.00 | 5.99 | 0.00 | 7.74 |
| 512 | 2.709270 | 34.119000 | 34.119000 | 34.119000 | 34.119000 | 0.00 | 0.00 | 0.00 | 0.00 | 0.00 | 0.00 | 0.00 | 0.00 | 0.00 | 0.40 | 0.00 | 0.00 |
| 1.024 | 3.010300 | 25.114000 | 25.114000 | 25.114000 | 25.114000 | 0.00 | 0.00 | 0.00 | 0.00 | 0.00 | 0.00 | 0.00 | 0.00 | 0.00 | 0.00 | 0.00 | 0.00 |
| 2048 | 3.311330 | 24.772000 | 24.772000 | 24.772000 | 24.772000 | 0.00 | 0.00 | 0.00 | 0.00 | 0.00 | 0.00 | 0.00 | 0.00 | 0.00 | 0.00 | 0.00 | 0.00 |
| 4096 | 3.612360 | 16.831000 | 16.831000 | 16.831000 | 16.831000 | 0.00 | 0.00 | 0.00 | 0.00 | 0.00 | 0.00 | 0.00 | 0.00 | 0.00 | 0.00 | 0.00 | 0.00 |
| 8182 | 3.912859 | 17.287000 | 17.287000 | 17.287000 | 17.287000 | 0.00 | 0.00 | 0.00 | 0.00 | 0.00 | 0.00 | 0.00 | 0.00 | 0.00 | 0.00 | 0.00 | 0.00 |

Figure 1E

|  | Concentraçao | Fluconazole (IC_50_ = 271.3 μg/mL) | | | | AP1 (IC_50_ = 1621.8 μg/mL) | | | | AP2 (IC_50_ = 345.5 μg/mL) | | | | AP3 (IC_50_ = 1949.8 μg/mL) | | | |
| --- | --- | --- | --- | --- | --- | --- | --- | --- | --- | --- | --- | --- | --- | --- | --- | --- | --- |
| 0 | 0.000000 | 100.000000 | 100.000000 | 100.000000 | 100.000000 | 100.000000 | 100.000000 | 100.000000 | 100.000000 | 100.000000 | 100.000000 | 100.000000 | 100.000000 | 100.000000 | 100.000000 | 100.000000 | 100.000000 |
| 8 | 0.903090 | 64.687000 | 64.687000 | 64.687000 | 64.687000 | 78.090000 | 97.850000 | 70.780000 | 87.180000 | 88.700000 | 89.340000 | 69.040000 | 58.150000 | 91.300000 | 88.720000 | 80.170000 | 77.580000 |
| 16 | 1.204120 | 64.541000 | 64.541000 | 64.541000 | 64.541000 | 63.460000 | 55.360000 | 73.340000 | 80.460000 | 89.850000 | 71.690000 | 64.640000 | 66.560000 | 78.880000 | 81.060000 | 72.910000 | 85.040000 |
| 32 | 1.505150 | 63.302000 | 63.302000 | 63.302000 | 63.302000 | 64.160000 | 90.640000 | 64.750000 | 68.310000 | 67.150000 | 75.690000 | 75.910000 | 62.020000 | 103.030000 | 79.770000 | 76.790000 | 76.790000 |
| 64 | 1.806180 | 59.257000 | 59.257000 | 59.257000 | 59.257000 | 52.820000 | 59.930000 | 67.050000 | 73.960000 | 65.600000 | 59.400000 | 62.610000 | 85.260000 | 79.000000 | 100.070000 | 86.360000 | 59.720000 |
| 128 | 2.107210 | 57.034000 | 57.034000 | 57.034000 | 57.034000 | 58.620000 | 88.460000 | 76.800000 | 66.530000 | 71.660000 | 88.110000 | 78.500000 | 77.640000 | 97.940000 | 84.820000 | 81.830000 | 74.680000 |
| 256 | 2.408240 | 51.446000 | 51.446000 | 51.446000 | 51.446000 | 54.990000 | 68.820000 | 56.770000 | 85.030000 | 51.500000 | 80.560000 | 89.100000 | 66.450000 | 76.340000 | 83.900000 | 60.440000 | 83.100000 |
| 512 | 2.709270 | 0.000000 | 0.000000 | 0.000000 | 0.000000 | 63.560000 | 67.710000 | 68.110000 | 67.710000 | 46.470000 | 6.090000 | 57.370000 | 42.630000 | 77.630000 | 78.230000 | 60.540000 | 79.030000 |
| 1.024 | 3.010300 | 0.000000 | 0.000000 | 0.000000 | 0.000000 | 69.190000 | 74.930000 | 70.970000 | 72.750000 | 2.380000 | -0.830000 | 1.520000 | 5.150000 | 73.010000 | 57.900000 | 69.040000 | 77.190000 |
| 2048 | 3.311330 | 0.000000 | 0.000000 | 0.000000 | 0.000000 | 45.780000 | 47.550000 | 70.870000 | 51.310000 | -2.060000 | 1.360000 | 3.500000 | 3.070000 | 65.360000 | 77.090000 | 53.630000 | 66.350000 |
| 4096 | 3.612360 | 0.000000 | 0.000000 | 0.000000 | 0.000000 | 22.080000 | 0.000000 | 10.620000 | 9.630000 | -3.150000 | 2.620000 | 2.830000 | 2.400000 | 5.770000 | 13.720000 | 4.770000 | -0.600000 |
| 8182 | 3.912859 | 0.000000 | 0.000000 | 0.000000 | 0.000000 | 0.000000 | 0.000000 | 0.000000 | 1.880000 | 0.000000 | 3.660000 | 1.310000 | 1.100000 | 4.420000 | 3.030000 | 6.010000 | 3.830000 |

Figure 1F

|  | Concentraçao | Fluconazole (IC_50_ = 271.0 μg/mL) | | | | AP1 (IC_50_ = 44.4 μg/mL) | | | | AP2 (IC_50_ = 32.4 μg/mL) | | | | AP3 (IC_50_ = 45.2 μg/mL) | | | |
| --- | --- | --- | --- | --- | --- | --- | --- | --- | --- | --- | --- | --- | --- | --- | --- | --- | --- |
| 0 | 0.000000 | 100.000000 | 100.000000 | 100.000000 | 100.000000 | 101.74 | 84.62 | 100.00 | 113.65 | 105.63 | 100.00 | 90.71 | 103.66 | 100.00 | 100.00 | 103.62 | 96.38 |
| 8 | 0.903090 | 64.687000 | 64.687000 | 64.687000 | 64.687000 | 68.61 | 76.05 | 89.45 | 92.18 | 60.13 | 64.64 | 90.17 | 64.84 | 77.66 | 73.13 | 72.68 | 74.49 |
| 16 | 1.204120 | 64.541000 | 64.541000 | 64.541000 | 64.541000 | 74.26 | 71.77 | 74.26 | 73.43 | 63.27 | 82.71 | 75.64 | 73.87 | 87.33 | 86.88 | 78.96 | 91.86 |
| 32 | 1.505150 | 63.302000 | 63.302000 | 63.302000 | 63.302000 | 96.40 | 78.29 | 57.20 | 77.30 | 54.34 | 49.23 | 57.28 | 47.86 | 68.55 | 57.24 | 58.60 | 50.00 |
| 64 | 1.806180 | 59.257000 | 59.257000 | 59.257000 | 59.257000 | 33.81 | 43.07 | 53.91 | 41.50 | 45.21 | 64.84 | 54.04 | 48.15 | 64.25 | 65.16 | 64.25 | 64.56 |
| 128 | 2.107210 | 57.034000 | 57.034000 | 57.034000 | 57.034000 | 18.80 | 7.46 | 1.18 | 2.42 | 0.00 | 0.00 | 0.00 | 2.85 | 0.00 | 0.00 | 0.00 | 7.52 |
| 256 | 2.408240 | 51.446000 | 51.446000 | 51.446000 | 51.446000 | 6.51 | 0.00 | 0.00 | 0.00 | 0.00 | 0.00 | 0.00 | 0.00 | 0.00 | 0.00 | 0.00 | 0.00 |
| 512 | 2.709270 | 0.000000 | 0.000000 | 0.000000 | 0.000000 | 0.00 | 0.00 | 0.00 | 0.00 | 0.00 | 0.00 | 0.00 | 0.00 | 0.00 | 0.00 | 0.00 | 0.00 |
| 1.024 | 3.010300 | 0.000000 | 0.000000 | 0.000000 | 0.000000 | 0.00 | 0.00 | 0.00 | 0.00 | 0.00 | 0.00 | 0.00 | 0.00 | 0.00 | 0.00 | 0.00 | 0.00 |
| 2048 | 3.311330 | 0.000000 | 0.000000 | 0.000000 | 0.000000 | 0.00 | 0.00 | 0.00 | 0.00 | 0.00 | 0.00 | 0.00 | 0.00 | 0.00 | 0.00 | 0.00 | 0.00 |
| 4096 | 3.612360 | 0.000000 | 0.000000 | 0.000000 | 0.000000 | 0.00 | 0.00 | 0.00 | 0.00 | 0.00 | 0.00 | 0.00 | 0.00 | 0.00 | 0.00 | 0.00 | 0.00 |
| 8182 | 3.912859 | 0.000000 | 0.000000 | 0.000000 | 0.000000 | 0.00 | 0.00 | 0.00 | 0.00 | 0.00 | 0.00 | 0.00 | 0.00 | 0.00 | 0.00 | 0.00 | 0.00 |
